# (3*Z*)-1,1,1-Trifluoro-4-phenyl-4-[(2-{[(1*Z*)-4,4,4-trifluoro-3-oxo-1-phenyl­but-1-en-1-yl]amino}­eth­yl)amino]­but-3-en-2-one

**DOI:** 10.1107/S1600536812028875

**Published:** 2012-06-30

**Authors:** Abdullah M. Asiri, Hassan M. Faidallah, Khalid A. Alamry, Seik Weng Ng, Edward R. T. Tiekink

**Affiliations:** a Center of Excellence for Advanced Materials Research (CEAMR), King Abdulaziz University, PO Box 80203, Jeddah 21589, Saudi Arabia; b Chemistry Department, Faculty of Science, King Abdulaziz University, PO Box 80203, Jeddah 21589, Saudi Arabia; c Department of Chemistry, University of Malaya, 50603 Kuala Lumpur, Malaysia

## Abstract

In the title compound, C_22_H_18_F_6_N_2_O_2_, the five atoms comprising each O=C—C=C—N fragment are almost coplanar (the r.m.s. deviation for the fitted atoms being 0.008 and 0.002 Å) and form a dihedral angle of 47.70 (12)°. The phenyl ring attached to each of the O=C—C=C—N fragments is twisted out of the respective plane with dihedral angles of 64.46 (11) and 61.82 (10)°, respectively. An almost orthogonal relationship for the phenyl rings is indicated by the dihedral angle between them of 78.19 (14)°. The conformation about each ethyl­ene bond is *Z*, which allows for the formation of intra­molecular N—H⋯O hydrogen bonds which close *S*(6) loops. The most prominent feature of the crystal packing are N—H⋯O hydrogen bonds that result in supra­molecular chains along the *a* axis. The F atoms of one –CF_3_ groups are disordered over three sets of sites with site-occupation factors of 0.318 (4), 0.360 (10) and 0.322 (9).

## Related literature

For the structure of the compound in which the CF_3_ substituents of the title compound are replaced by 2-thienyl groups, see: Asiri *et al.* (2011 ▶).
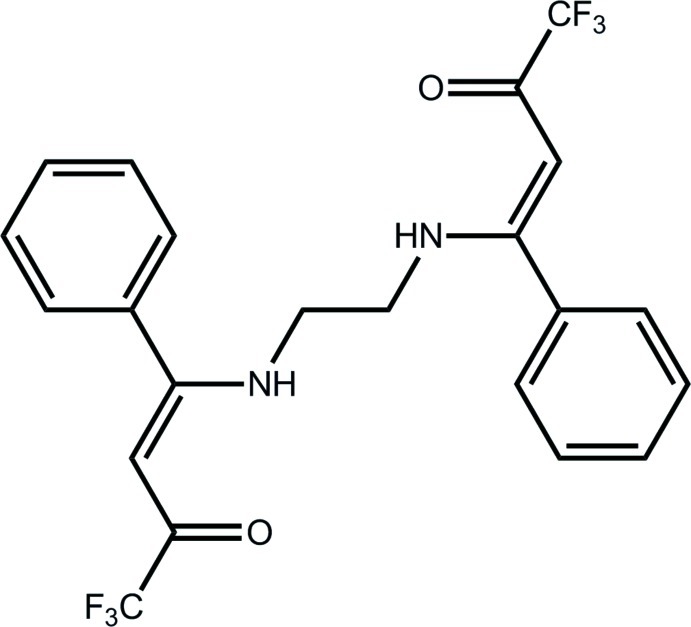


## Experimental

### 

#### Crystal data

C_22_H_18_F_6_N_2_O_2_
*M* *_r_* = 456.38
Monoclinic, 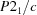

*a* = 13.0411 (9) Å
*b* = 15.897 (1) Å
*c* = 10.9417 (9) Å
β = 112.306 (9)°
*V* = 2098.6 (3) Å^3^
*Z* = 4
Mo *K*α radiation
μ = 0.13 mm^−1^
*T* = 100 K
0.35 × 0.15 × 0.15 mm

#### Data collection

Agilent SuperNova Dual diffractometer with an Atlas detector
Absorption correction: multi-scan (*CrysAlis PRO*; Agilent, 2012 ▶) *T*_min_ = 0.538, *T*_max_ = 1.000
10523 measured reflections
4845 independent reflections
3146 reflections with *I* > 2σ(*I*)
*R*_int_ = 0.041

#### Refinement

*R*[*F* ^2^ > 2σ(*F* ^2^)] = 0.060
*wR*(*F* ^2^) = 0.165
*S* = 1.02
4845 reflections
309 parameters
19 restraints
H atoms treated by a mixture of independent and constrained refinement
Δρ_max_ = 0.51 e Å^−3^
Δρ_min_ = −0.50 e Å^−3^

### 

Data collection: *CrysAlis PRO* (Agilent, 2012 ▶); cell refinement: *CrysAlis PRO*; data reduction: *CrysAlis PRO*; program(s) used to solve structure: *SHELXS97* (Sheldrick, 2008 ▶); program(s) used to refine structure: *SHELXL97* (Sheldrick, 2008 ▶); molecular graphics: *ORTEP-3* (Farrugia, 1997 ▶) and *DIAMOND* (Brandenburg, 2006 ▶); software used to prepare material for publication: *publCIF* (Westrip, 2010 ▶).

## Supplementary Material

Crystal structure: contains datablock(s) global, I. DOI: 10.1107/S1600536812028875/bt5954sup1.cif

Structure factors: contains datablock(s) I. DOI: 10.1107/S1600536812028875/bt5954Isup2.hkl

Supplementary material file. DOI: 10.1107/S1600536812028875/bt5954Isup3.cml

Additional supplementary materials:  crystallographic information; 3D view; checkCIF report

## Figures and Tables

**Table 1:** Hydrogen-bond geometry (Å, °)

*D*—H⋯*A*	*D*—H	H⋯*A*	*D*⋯*A*	*D*—H⋯*A*
N1—H1⋯O1	0.91 (3)	2.02 (3)	2.719 (3)	133 (3)
N1—H1⋯O1^i^	0.91 (3)	2.28 (3)	2.997 (3)	135 (3)
N2—H2⋯O2	0.88 (3)	2.02 (3)	2.709 (3)	134 (3)
N2—H2⋯O2^ii^	0.88 (3)	2.33 (3)	3.039 (3)	137 (3)

Symmetry codes: (i) 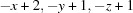; (ii) 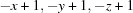.

**Fig. 1.**
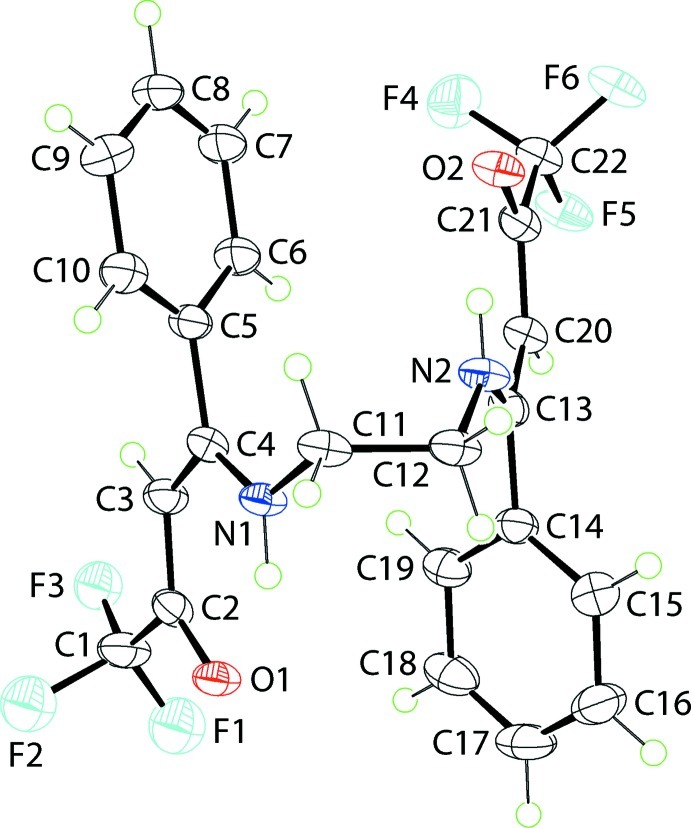
The molecular structure of (I) showing the atom-labelling scheme and displacement ellipsoids at the 50% probability level. The C1—CF3 group is disordered over three positions. The orientation with a site occupancy factor = 0.318 (4) is illustrated here.

**Fig. 2.**
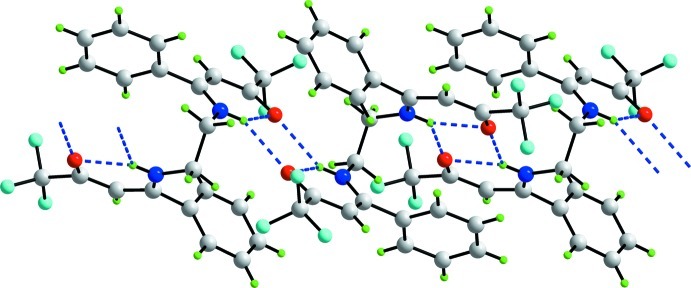
A view of the supramolecular chain along the a axis in (I) mediated by N—H···O hydrogen bonds shown as blue dashed lines.

## supplementary crystallographic information

### Comment

Recently, some of us described the structure of the 2-thienyl derivative (Asiri *et al.*, 2011) of the title compound, (I). Herein, the crystal and molecular structure of (I) is described which has –CF_3_ groups rather than thienyl substituents.

In (I), Fig. 1, the five atoms comprising each O═C—C═C—N fragment are co-planar with the r.m.s. deviation for the fitted atoms being 0.008 Å [for the plane containing the O1 atom] and 0.002 Å [O2]; the dihedral angle between the planes is 47.70 (12)°. The conformation about each ethylene bond is *Z* allowing for the formation of intramolecular N—H···O hydrogen bonds which close *S*(6) loops, Table 1; a similar conformation and *S*(6) loops were observed in the two independent molecules of the 2-thienyl derivative (Asiri *et al.*, 2011). The attached phenyl ring is twisted out of the plane through the O═C—C═C—N fragment, forming dihedral angles of 64.46 (11) and 61.82 (10)°, respectively; the dihedral angle between the phenyl rings is 78.19 (14)°.

The crystal packing also features N—H···O hydrogen bonds so that each amine-H and each carbonyl-O atom is bifurcated, Table 1. The result is the formation of four-membered {···H···O}_2_ synthons and supramolecular chains along the *a* axis, Fig. 2.

### Experimental

A mixture of the *N*,*N*'-bis(1-ethylidene)ethane-1,2-diamine (0.01 *M*) in THF (30 ml) and trifluroacetic anhydride (0.025 *M*) was refluxed for 2 h. The solid which separated on cooling was recrystallized from ethanol. *M*. pt: 477–478 K. Yield: 70%.

### Refinement

Carbon-bound H-atoms were placed in calculated positions [C—H = 0.95–0.99 Å, *U*_iso_(H) = 1.2*U*_eq_(C)] and were included in the refinement in the riding model approximation. The N-bound H-atoms were located in a difference were refined freely. One trifluoromethyl group is disordered over three positions in respect to the F atoms. The C—F distances were restrained to within 1.35±0.01 Å, and the F···F distances to 2.21±0.01 Å. The disordered F atoms were refined isotropically and the final site occupancies were 0.318 (4), 0.360 (10) and 0.322 (9) for the unprimed, primed and doubly primed atoms, respectively.

### Crystal data

**Table:**

C_22_H_18_F_6_N_2_O_2_	*F*(000) = 936
*M**_r_* = 456.38	*D*_x_ = 1.444 Mg m^−^^3^
Monoclinic, *P*2_1_/*c*	Mo *K*α radiation, λ = 0.71073 Å
Hall symbol: -P 2ybc	Cell parameters from 2319 reflections
*a* = 13.0411 (9) Å	θ = 2.4–27.5°
*b* = 15.897 (1) Å	µ = 0.13 mm^−^^1^
*c* = 10.9417 (9) Å	*T* = 100 K
β = 112.306 (9)°	Prism, colourless
*V* = 2098.6 (3) Å^3^	0.35 × 0.15 × 0.15 mm
*Z* = 4	

### Data collection

**Table:**

Agilent SuperNova Dual diffractometer with an Atlas detector	4845 independent reflections
Radiation source: SuperNova (Mo) X-ray Source	3146 reflections with *I* > 2σ(*I*)
Mirror monochromator	*R*_int_ = 0.041
Detector resolution: 10.4041 pixels mm^-1^	θ_max_ = 27.6°, θ_min_ = 2.4°
ω scan	*h* = −16→16
Absorption correction: multi-scan (*CrysAlis PRO*; Agilent, 2012)	*k* = −15→20
*T*_min_ = 0.538, *T*_max_ = 1.000	*l* = −10→14
10523 measured reflections	

### Refinement

**Table:**

Refinement on *F*^2^	Primary atom site location: structure-invariant direct methods
Least-squares matrix: full	Secondary atom site location: difference Fourier map
*R*[*F*^2^ > 2σ(*F*^2^)] = 0.060	Hydrogen site location: inferred from neighbouring sites
*wR*(*F*^2^) = 0.165	H atoms treated by a mixture of independent and constrained refinement
*S* = 1.02	*w* = 1/[σ^2^(*F*_o_^2^) + (0.0641*P*)^2^ + 1.085*P*] where *P* = (*F*_o_^2^ + 2*F*_c_^2^)/3
4845 reflections	(Δ/σ)_max_ = 0.002
309 parameters	Δρ_max_ = 0.51 e Å^−^^3^
19 restraints	Δρ_min_ = −0.50 e Å^−^^3^

### Special details

**Table:**

Geometry. All e.s.d.'s (except the e.s.d. in the dihedral angle between two l.s. planes) are estimated using the full covariance matrix. The cell e.s.d.'s are taken into account individually in the estimation of e.s.d.'s in distances, angles and torsion angles; correlations between e.s.d.'s in cell parameters are only used when they are defined by crystal symmetry. An approximate (isotropic) treatment of cell e.s.d.'s is used for estimating e.s.d.'s involving l.s. planes.
Refinement. Refinement of *F*^2^ against ALL reflections. The weighted *R*-factor *wR* and goodness of fit *S* are based on *F*^2^, conventional *R*-factors *R* are based on *F*, with *F* set to zero for negative *F*^2^. The threshold expression of *F*^2^ > σ(*F*^2^) is used only for calculating *R*-factors(gt) *etc*. and is not relevant to the choice of reflections for refinement. *R*-factors based on *F*^2^ are statistically about twice as large as those based on *F*, and *R*- factors based on ALL data will be even larger.

### Fractional atomic coordinates and isotropic or equivalent isotropic displacement parameters (Å^2^)

**Table:**

	*x*	*y*	*z*	*U*_iso_*/*U*_eq_	Occ. (<1)
O1	1.01821 (13)	0.40373 (11)	0.50828 (17)	0.0287 (4)	
O2	0.44440 (13)	0.44780 (12)	0.37678 (17)	0.0304 (4)	
N2	0.63608 (17)	0.52490 (14)	0.3995 (2)	0.0276 (5)	
N1	0.86126 (16)	0.46861 (13)	0.5887 (2)	0.0253 (5)	
F1	1.0661 (7)	0.2668 (4)	0.3981 (6)	0.0556 (19)*	0.318 (4)
F2	1.1419 (5)	0.2588 (4)	0.6132 (6)	0.0514 (19)*	0.318 (4)
F3	0.9898 (5)	0.1853 (4)	0.4922 (7)	0.041 (2)*	0.318 (4)
F1'	1.0158 (7)	0.2543 (4)	0.3630 (4)	0.0332 (16)*	0.360 (10)
F2'	1.1461 (3)	0.2603 (3)	0.5612 (7)	0.0142 (13)*	0.360 (10)
F3'	1.0020 (5)	0.1831 (3)	0.5290 (9)	0.0150 (14)*	0.360 (10)
F1"	0.9793 (7)	0.2360 (5)	0.3631 (5)	0.055 (2)*	0.322 (9)
F2"	1.1364 (4)	0.2625 (3)	0.5098 (9)	0.0290 (17)*	0.322 (9)
F3"	1.0224 (6)	0.1860 (4)	0.5657 (8)	0.038 (2)*	0.322 (9)
F4	0.35986 (14)	0.28737 (11)	0.28834 (17)	0.0491 (5)	
F5	0.37037 (12)	0.31466 (10)	0.10070 (16)	0.0394 (4)	
F6	0.26345 (11)	0.38801 (10)	0.16715 (15)	0.0382 (4)	
C1	1.0361 (2)	0.25715 (17)	0.4985 (3)	0.0370 (7)	
C2	0.98029 (19)	0.33504 (16)	0.5274 (2)	0.0260 (6)	
C3	0.8934 (2)	0.32256 (17)	0.5709 (3)	0.0284 (6)	
H3	0.8712	0.2666	0.5791	0.034*	
C4	0.83811 (19)	0.38913 (16)	0.6026 (2)	0.0252 (5)	
C5	0.75063 (19)	0.36831 (17)	0.6547 (3)	0.0277 (6)	
C6	0.6563 (2)	0.32548 (17)	0.5745 (3)	0.0314 (6)	
H6	0.6480	0.3097	0.4875	0.038*	
C7	0.5742 (2)	0.30574 (19)	0.6215 (3)	0.0379 (7)	
H7	0.5091	0.2773	0.5660	0.045*	
C8	0.5872 (2)	0.3275 (2)	0.7495 (3)	0.0409 (7)	
H8	0.5310	0.3138	0.7815	0.049*	
C9	0.6812 (2)	0.3689 (2)	0.8300 (3)	0.0413 (7)	
H9	0.6902	0.3832	0.9178	0.050*	
C10	0.7632 (2)	0.38983 (19)	0.7832 (3)	0.0347 (7)	
H10	0.8278	0.4188	0.8388	0.042*	
C11	0.80105 (19)	0.54221 (16)	0.6068 (3)	0.0274 (6)	
H11A	0.8550	0.5849	0.6595	0.033*	
H11B	0.7551	0.5255	0.6569	0.033*	
C12	0.72688 (19)	0.58088 (16)	0.4758 (3)	0.0279 (6)	
H12A	0.6956	0.6343	0.4930	0.033*	
H12B	0.7720	0.5941	0.4232	0.033*	
C13	0.62859 (19)	0.47929 (17)	0.2947 (2)	0.0265 (6)	
C14	0.71669 (19)	0.48676 (17)	0.2393 (2)	0.0271 (6)	
C15	0.7362 (2)	0.56297 (19)	0.1885 (3)	0.0369 (7)	
H15	0.6925	0.6110	0.1874	0.044*	
C16	0.8199 (2)	0.5682 (2)	0.1397 (3)	0.0437 (8)	
H16	0.8335	0.6201	0.1053	0.052*	
C17	0.8833 (2)	0.4990 (2)	0.1406 (3)	0.0440 (8)	
H17	0.9409	0.5034	0.1079	0.053*	
C18	0.8634 (2)	0.4231 (2)	0.1890 (3)	0.0396 (7)	
H18	0.9074	0.3754	0.1896	0.047*	
C19	0.7791 (2)	0.41635 (19)	0.2368 (3)	0.0315 (6)	
H19	0.7642	0.3637	0.2678	0.038*	
C20	0.54108 (19)	0.42385 (16)	0.2348 (2)	0.0267 (6)	
H20	0.5397	0.3925	0.1603	0.032*	
C21	0.45487 (19)	0.41255 (16)	0.2804 (2)	0.0263 (6)	
C22	0.3625 (2)	0.35051 (17)	0.2078 (3)	0.0302 (6)	
H1	0.918 (3)	0.479 (2)	0.562 (3)	0.054 (10)*	
H2	0.583 (3)	0.5189 (19)	0.430 (3)	0.046 (9)*	

### Atomic displacement parameters (Å^2^)

**Table:**

	*U*^11^	*U*^22^	*U*^33^	*U*^12^	*U*^13^	*U*^23^
O1	0.0229 (9)	0.0269 (10)	0.0412 (10)	−0.0004 (8)	0.0176 (8)	0.0006 (8)
O2	0.0222 (8)	0.0362 (11)	0.0367 (10)	−0.0024 (8)	0.0155 (8)	−0.0076 (9)
N2	0.0201 (10)	0.0286 (12)	0.0379 (12)	−0.0047 (9)	0.0155 (9)	−0.0050 (10)
N1	0.0197 (10)	0.0252 (12)	0.0355 (11)	−0.0005 (9)	0.0156 (9)	−0.0017 (10)
F4	0.0464 (10)	0.0404 (10)	0.0557 (11)	−0.0172 (9)	0.0140 (9)	0.0040 (9)
F5	0.0262 (8)	0.0437 (10)	0.0501 (9)	−0.0018 (7)	0.0167 (7)	−0.0203 (8)
F6	0.0196 (7)	0.0469 (10)	0.0478 (9)	0.0001 (7)	0.0125 (7)	−0.0173 (8)
C1	0.0284 (14)	0.0281 (15)	0.0609 (19)	−0.0017 (12)	0.0241 (14)	0.0029 (14)
C2	0.0188 (11)	0.0237 (14)	0.0340 (13)	0.0021 (10)	0.0085 (10)	0.0008 (11)
C3	0.0231 (12)	0.0240 (14)	0.0407 (14)	−0.0012 (11)	0.0148 (11)	0.0035 (12)
C4	0.0172 (11)	0.0297 (14)	0.0278 (12)	−0.0022 (11)	0.0075 (10)	0.0006 (11)
C5	0.0204 (11)	0.0289 (14)	0.0366 (13)	0.0010 (11)	0.0139 (11)	0.0059 (12)
C6	0.0244 (12)	0.0327 (15)	0.0378 (14)	−0.0052 (12)	0.0126 (11)	0.0031 (12)
C7	0.0247 (13)	0.0413 (17)	0.0482 (16)	−0.0053 (13)	0.0144 (12)	0.0106 (14)
C8	0.0283 (14)	0.0485 (19)	0.0539 (18)	0.0021 (14)	0.0245 (14)	0.0166 (15)
C9	0.0376 (15)	0.056 (2)	0.0385 (15)	−0.0001 (15)	0.0235 (13)	0.0081 (15)
C10	0.0254 (13)	0.0469 (18)	0.0321 (14)	−0.0035 (13)	0.0114 (11)	0.0032 (13)
C11	0.0224 (12)	0.0255 (14)	0.0392 (14)	−0.0042 (11)	0.0170 (11)	−0.0078 (12)
C12	0.0230 (12)	0.0236 (13)	0.0419 (14)	−0.0031 (11)	0.0178 (11)	−0.0036 (12)
C13	0.0218 (12)	0.0253 (14)	0.0340 (13)	0.0052 (11)	0.0123 (11)	0.0027 (11)
C14	0.0214 (12)	0.0322 (14)	0.0287 (13)	−0.0039 (11)	0.0105 (10)	−0.0007 (11)
C15	0.0333 (14)	0.0386 (17)	0.0404 (15)	−0.0017 (13)	0.0159 (13)	0.0055 (13)
C16	0.0382 (16)	0.058 (2)	0.0377 (15)	−0.0148 (16)	0.0173 (13)	0.0064 (15)
C17	0.0307 (14)	0.071 (2)	0.0371 (16)	−0.0130 (16)	0.0208 (13)	−0.0074 (16)
C18	0.0277 (14)	0.057 (2)	0.0376 (15)	0.0010 (14)	0.0161 (12)	−0.0115 (14)
C19	0.0263 (13)	0.0392 (16)	0.0314 (13)	−0.0011 (12)	0.0136 (11)	−0.0063 (12)
C20	0.0213 (12)	0.0282 (14)	0.0313 (13)	−0.0007 (11)	0.0109 (10)	−0.0032 (11)
C21	0.0206 (12)	0.0248 (13)	0.0333 (13)	0.0002 (11)	0.0099 (11)	−0.0011 (11)
C22	0.0226 (12)	0.0306 (15)	0.0385 (14)	−0.0002 (11)	0.0129 (11)	−0.0045 (12)

### Geometric parameters (Å, º)

**Table:**

O1—C2	1.249 (3)	C7—C8	1.389 (4)
O2—C21	1.247 (3)	C7—H7	0.9500
N2—C13	1.328 (3)	C8—C9	1.376 (4)
N2—C12	1.463 (3)	C8—H8	0.9500
N2—H2	0.88 (3)	C9—C10	1.390 (4)
N1—C4	1.321 (3)	C9—H9	0.9500
N1—C11	1.464 (3)	C10—H10	0.9500
N1—H1	0.91 (3)	C11—C12	1.521 (4)
F1—C1	1.308 (5)	C11—H11A	0.9900
F2—C1	1.471 (5)	C11—H11B	0.9900
F3—C1	1.282 (6)	C12—H12A	0.9900
F1'—C1	1.405 (5)	C12—H12B	0.9900
F2'—C1	1.335 (4)	C13—C20	1.393 (4)
F3'—C1	1.345 (5)	C13—C14	1.493 (3)
F1"—C1	1.422 (6)	C14—C19	1.390 (4)
F2"—C1	1.269 (5)	C14—C15	1.396 (4)
F3"—C1	1.397 (5)	C15—C16	1.388 (4)
F4—C22	1.344 (3)	C15—H15	0.9500
F5—C22	1.342 (3)	C16—C17	1.374 (4)
F6—C22	1.336 (3)	C16—H16	0.9500
C1—C2	1.529 (4)	C17—C18	1.382 (4)
C2—C3	1.400 (3)	C17—H17	0.9500
C3—C4	1.397 (4)	C18—C19	1.389 (4)
C3—H3	0.9500	C18—H18	0.9500
C4—C5	1.493 (3)	C19—H19	0.9500
C5—C6	1.388 (4)	C20—C21	1.404 (3)
C5—C10	1.395 (4)	C20—H20	0.9500
C6—C7	1.388 (4)	C21—C22	1.526 (4)
C6—H6	0.9500		
			
C13—N2—C12	127.1 (2)	C9—C10—C5	120.0 (3)
C13—N2—H2	117 (2)	C9—C10—H10	120.0
C12—N2—H2	116 (2)	C5—C10—H10	120.0
C4—N1—C11	126.2 (2)	N1—C11—C12	112.2 (2)
C4—N1—H1	118 (2)	N1—C11—H11A	109.2
C11—N1—H1	116 (2)	C12—C11—H11A	109.2
F3—C1—F1	110.2 (4)	N1—C11—H11B	109.2
F2'—C1—F3'	107.3 (4)	C12—C11—H11B	109.2
F2"—C1—F3"	109.6 (4)	H11A—C11—H11B	107.9
F2'—C1—F1'	106.2 (3)	N2—C12—C11	112.3 (2)
F3'—C1—F1'	106.6 (4)	N2—C12—H12A	109.1
F2"—C1—F1"	103.1 (4)	C11—C12—H12A	109.1
F3"—C1—F1"	103.7 (4)	N2—C12—H12B	109.1
F3—C1—F2	109.4 (4)	C11—C12—H12B	109.1
F1—C1—F2	103.4 (4)	H12A—C12—H12B	107.9
F2"—C1—C2	118.6 (3)	N2—C13—C20	122.0 (2)
F3—C1—C2	118.8 (4)	N2—C13—C14	119.4 (2)
F1—C1—C2	113.3 (4)	C20—C13—C14	118.6 (2)
F2'—C1—C2	111.5 (3)	C19—C14—C15	119.6 (2)
F3'—C1—C2	115.5 (3)	C19—C14—C13	119.4 (2)
F3"—C1—C2	112.4 (3)	C15—C14—C13	121.0 (2)
F1'—C1—C2	109.3 (3)	C16—C15—C14	119.7 (3)
F1"—C1—C2	107.8 (3)	C16—C15—H15	120.2
F2—C1—C2	100.1 (3)	C14—C15—H15	120.2
O1—C2—C3	127.2 (2)	C17—C16—C15	120.5 (3)
O1—C2—C1	115.1 (2)	C17—C16—H16	119.8
C3—C2—C1	117.7 (2)	C15—C16—H16	119.8
C4—C3—C2	122.6 (2)	C16—C17—C18	120.2 (3)
C4—C3—H3	118.7	C16—C17—H17	119.9
C2—C3—H3	118.7	C18—C17—H17	119.9
N1—C4—C3	122.3 (2)	C17—C18—C19	120.2 (3)
N1—C4—C5	119.8 (2)	C17—C18—H18	119.9
C3—C4—C5	117.9 (2)	C19—C18—H18	119.9
C6—C5—C10	119.6 (2)	C14—C19—C18	119.9 (3)
C6—C5—C4	119.3 (2)	C14—C19—H19	120.1
C10—C5—C4	121.1 (2)	C18—C19—H19	120.1
C7—C6—C5	120.0 (3)	C13—C20—C21	122.5 (2)
C7—C6—H6	120.0	C13—C20—H20	118.7
C5—C6—H6	120.0	C21—C20—H20	118.7
C6—C7—C8	120.1 (3)	O2—C21—C20	127.1 (2)
C6—C7—H7	120.0	O2—C21—C22	114.4 (2)
C8—C7—H7	120.0	C20—C21—C22	118.6 (2)
C9—C8—C7	120.2 (2)	F6—C22—F5	106.5 (2)
C9—C8—H8	119.9	F6—C22—F4	106.8 (2)
C7—C8—H8	119.9	F5—C22—F4	106.5 (2)
C8—C9—C10	120.1 (3)	F6—C22—C21	110.9 (2)
C8—C9—H9	119.9	F5—C22—C21	114.9 (2)
C10—C9—H9	119.9	F4—C22—C21	110.7 (2)
			
F2"—C1—C2—O1	27.6 (6)	C7—C8—C9—C10	0.6 (5)
F3—C1—C2—O1	−167.4 (4)	C8—C9—C10—C5	−0.5 (5)
F1—C1—C2—O1	−35.7 (5)	C6—C5—C10—C9	−0.4 (4)
F2'—C1—C2—O1	52.0 (4)	C4—C5—C10—C9	−179.2 (3)
F3'—C1—C2—O1	174.8 (5)	C4—N1—C11—C12	−104.0 (3)
F3"—C1—C2—O1	157.3 (4)	C13—N2—C12—C11	−103.8 (3)
F1'—C1—C2—O1	−65.0 (5)	N1—C11—C12—N2	66.1 (3)
F1"—C1—C2—O1	−89.0 (5)	C12—N2—C13—C20	175.8 (2)
F2—C1—C2—O1	73.7 (4)	C12—N2—C13—C14	−3.7 (4)
F2"—C1—C2—C3	−152.8 (5)	N2—C13—C14—C19	118.6 (3)
F3—C1—C2—C3	12.2 (5)	C20—C13—C14—C19	−60.9 (3)
F1—C1—C2—C3	144.0 (4)	N2—C13—C14—C15	−62.2 (3)
F2'—C1—C2—C3	−128.3 (4)	C20—C13—C14—C15	118.2 (3)
F3'—C1—C2—C3	−5.5 (5)	C19—C14—C15—C16	−1.9 (4)
F3"—C1—C2—C3	−23.1 (5)	C13—C14—C15—C16	179.0 (2)
F1'—C1—C2—C3	114.7 (5)	C14—C15—C16—C17	0.2 (4)
F1"—C1—C2—C3	90.7 (5)	C15—C16—C17—C18	0.7 (4)
F2—C1—C2—C3	−106.6 (4)	C16—C17—C18—C19	0.1 (4)
O1—C2—C3—C4	−1.7 (4)	C15—C14—C19—C18	2.7 (4)
C1—C2—C3—C4	178.7 (2)	C13—C14—C19—C18	−178.2 (2)
C11—N1—C4—C3	173.6 (2)	C17—C18—C19—C14	−1.8 (4)
C11—N1—C4—C5	−6.8 (4)	N2—C13—C20—C21	0.3 (4)
C2—C3—C4—N1	2.8 (4)	C14—C13—C20—C21	179.8 (2)
C2—C3—C4—C5	−176.9 (2)	C13—C20—C21—O2	−0.7 (4)
N1—C4—C5—C6	116.6 (3)	C13—C20—C21—C22	179.8 (2)
C3—C4—C5—C6	−63.7 (3)	O2—C21—C22—F6	56.6 (3)
N1—C4—C5—C10	−64.5 (3)	C20—C21—C22—F6	−123.8 (3)
C3—C4—C5—C10	115.1 (3)	O2—C21—C22—F5	177.4 (2)
C10—C5—C6—C7	1.2 (4)	C20—C21—C22—F5	−3.0 (4)
C4—C5—C6—C7	−179.9 (2)	O2—C21—C22—F4	−61.8 (3)
C5—C6—C7—C8	−1.1 (4)	C20—C21—C22—F4	117.7 (3)
C6—C7—C8—C9	0.2 (5)		

### Hydrogen-bond geometry (Å, º)

**Table:**

*D*—H···*A*	*D*—H	H···*A*	*D*···*A*	*D*—H···*A*
N1—H1···O1	0.91 (3)	2.02 (3)	2.719 (3)	133 (3)
N1—H1···O1^i^	0.91 (3)	2.28 (3)	2.997 (3)	135 (3)
N2—H2···O2	0.88 (3)	2.02 (3)	2.709 (3)	134 (3)
N2—H2···O2^ii^	0.88 (3)	2.33 (3)	3.039 (3)	137 (3)

Symmetry codes: (i) −*x*+2, −*y*+1, −*z*+1; (ii) −*x*+1, −*y*+1, −*z*+1.
